# Filaggrin gene mutations and new SNPs in asthmatic patients: a cross-sectional study in a Spanish population

**DOI:** 10.1186/s13223-016-0137-x

**Published:** 2016-07-26

**Authors:** José Luis Cubero, María Isidoro-García, Nieves Segura, David Benito Pescador, Catalina Sanz, Félix Lorente, Ignacio Dávila, Carlos Colás

**Affiliations:** 1 Allergy Department, University Hospital Lozano Blesa of Zaragoza, Avda. San Juan Bosco 15. 50.009, Zaragoza, Spain; 2 Instituto de Investigación Sanitaria Aragón (IIS Aragón), Zaragoza, Spain; 3 Department of Clinical Biochemistry, University Hospital of Salamanca, Salamanca, Spain; 4 Instituto Biosanitario de Salamanca (IBSAL), Salamanca, Spain; 5 Department of Medicine, University of Salamanca, Salamanca, Spain; 6 Allergy Department, University Hospital of Salamanca, Salamanca, Spain; 7 Microbiology and Genetics Department, University of Salamanca, Salamanca, Spain; 8 Paediatrics Department, University Hospital of Salamanca, Salamanca, Spain; 9 Biomedical and Diagnostic Sciences Department, University of Salamanca, Salamanca, Spain

**Keywords:** Filaggrin, FLG, R501X, 2282del4, Asthma, SNPs, Atopy

## Abstract

**Background:**

Several null-mutations in the *FLG* gene that produce a decrease or absence of filaggrin in the skin and predispose to atopic dermatitis and *ichthyosis vulgaris* have been described. The relationship with asthma is less clear and may be due to the influence of atopy in patients with associated asthma.

**Methods:**

Four hundred individuals were included, 300 patients diagnosed with asthma divided into two groups according to their phenotype (allergic and non-allergic asthma) and 100 strictly characterized controls. The coding region and flanking regions of the *FLG* gene were amplified by PCR. We proceeded to the characterization of potential gene variants in that region by RFLP and sequencing and analysed their association with lung function parameters, asthma control and severity, and quality of life.

**Results:**

We identified two null-mutations (R501X and 2282del4), seven SNPs previously described in databases and three SNPs that had not been previously described. One of the SNP identified in this study (1741A > T) was more frequently detected in patients with non-allergic asthma, worse FVC, FEV1 and PEF values and a higher treatment step. In addition, lowered spirometric values were observed in the non-allergic group carrying any of the nonsynonymous SNPs.

**Conclusions:**

In the association study of genetic variants of the *FLG* gene in our population the 1741A > T polymorphism seems to be associated with non-allergic asthma.

## Background

“Atopic march” theory suggests that children with atopic predisposition may suffer various diseases throughout their life such as atopic dermatitis, rhinitis or asthma and can become sensitised to various allergens. With regard to aeroallergen sensitization it may manifest as allergic rhinoconjunctivitis and, in a proportion of subjects, as asthma [1].

Filaggrin is a key protein in the differentiation of the epidermis and the formation of the stratum corneum of the skin, the outermost layer of the skin, essential to prevent water loss through the epidermis and to avoid the entry of allergens, toxic chemicals and germs [2]. This protein is composed of 324 amino acids, has a molecular weight of 37 kDa and it corresponds to 6 % of the epidermis protein content. Filaggrin assists the binding of keratin filaments of the skin, hence its name (FILament AGGregation proteIN). Profilaggrin, which contains 10–12 tandemly repeated filaggrin units and is processed into filaggrin by specific dephosphorylation and proteolysis during terminal differentiation of the epidermal cells, is encoded by the *FLG* gene. This gene is located on the long arm of chromosome 1 at position 1q21.3, and it is located in the so-called EDC (epidermal differentiation complex) region, which brings together a group of more than 30 structural and evolutionarily related genes, which encode proteins of the epidermis, such as filaggrin, loricrin or involucrin, among others. Several studies using biopsies from various tissues had observed that filaggrin is also expressed in the oral mucosa, the tongue and in the nasal mucosa, where it is assumed that it also contributes to the barrier function [3–6]. Although in some studies protein expression could not be detected in nasal [4], oesophageal [4] or bronchial [7] mucosa, gene expression studies indexed in GeneCards^®^ (http://www.genecards.org) show that *FLG* gene expression was observed in lung and bronchial epithelium.

There are reports of various null-mutations in the *FLG* gene (R501X, 2282del4 and subsequently others) that produce a decrease or absence of filaggrin in the skin and predispose to *ichthyosis vulgaris* [8, 9] and atopic dermatitis [9–19]. It has been identified that heterozygous patients for one of the mutations have no discernible phenotype or may have a minor ichthyosis, while homozygous mutant and heterozygous combinations (heterozygous for the two mutations) show marked ichthyosis with an obvious histological defect of the skin barrier [8]. All these mutations and their relationship to asthma is not so clear [10–26] and it could be that the relationship found in some studies is due to the influence of eczema or atopic dermatitis in subjects who have asthma associated with these skin diseases [27, 28], although, for the moment, studies to assess their relationship in the phenotypes of allergic and non-allergic asthma [26] are scarce.

The aim of our study was to assess whether the association identified in some studies in connection with asthma might not be due directly to this disease but to any of the associated phenotypes and therefore we designed a study with two patient groups with well differentiated asthma phenotypes. In addition, we sought to assess the existence of other gene variants in that region, responsible for the association with some of the clinical and biological characteristics of patients with asthma.

## Methods

This was a descriptive, cross-sectional, observational study carried out in Spain.

### Ethics statement

All subjects included in the study were adults and gave their agreement by signing an informed consent. The study was approved by the local health authority and it´s Ethics Committee.

### Subjects

A total of 400 individuals were included: 300 Caucasian not related patients from the University Hospital Lozano Blesa (Zaragoza, Spain) diagnosed with asthma according to the GINA criteria (global initiative for asthma) [29] that came for consultations from July 2008 to August 2009 and 100 Spanish Caucasian controls from the University Hospital of Salamanca (Salamanca, Spain).

The required sample size calculation (statistical power of 80 % and significance level of 5 %) was calculated using the allelic frequencies obtained by Palmer et al. [21] eighty-five subjects in the control group (allelic frequency 0.01) and 256 patients in the asthma group (allelic frequency 0.10) for R501X null-mutation and 67 subjects in control group (allelic frequency 0.02) and 200 patients in asthma group (allelic frequency 0.14) for 2282del4 null-mutation.

Patients were divided into two groups with the following inclusion criteria:

- Non-allergic asthma (NA): 150 patients diagnosed with asthma (compatible clinical history, obstructive spirometry with a bronchodilator test with an increase of ≥12 % and ≥200 mL in FEV1 (forced expiratory volume in 1 s) or bronchial hyper-responsiveness test with methacholine), with negative skin tests with standard battery of common inhalant allergens in our environment and no symptoms upon exposure to allergens.
- Allergic asthma (AA): 150 patients diagnosed with asthma (same criteria as for NA), positive skin tests to any aeroallergen(s) in the standard battery for our environment and clinically relevant and symptoms upon exposure.

In addition, 100 healthy controls (HC) where chosen, who had to meet the following inclusion criteria: no history of atopy, asthma or chronic respiratory diseases, no family history of asthma or atopy and negative skin tests to a local airborne allergen battery.

The exclusion criteria were: age <18 years (to meet the local health authority and Ethics Committee legal requirements) or non-Caucasian or non-Spanish population.

### Variables analysed

Family history of atopy and personal history of atopic dermatitis, contact dermatitis, food allergy, drug allergy, hypersensitivity to non-steroidal anti-inflammatory drugs (NSAIDs), psoriasis or *ichthyosis vulgaris* were assessed. The presence of rhinitis, chronic rhinosinusitis, nasal polyps or anosmia was also evaluated.

Skin prick tests were performed with the battery of common airborne allergens in our environment (*Phleum pratense*, *Cynodon dactylon*, *Olea europaea*, *Parietaria judaica*, *Chenopodium album*, *Salsola kali*, *Platanus acerifolia*, *Cupressus sempervirens*, *Alternaria alternata*, *Cladosporium herbarum*, *Aspergillus fumigatus*, *Dermatophagoides pteronyssinus*, *Tyrophagus putrescentiae*, *Lepydoglyphus destructor* and dog and cat dander). Histamine hydrochloride (10 mg/mL) and glycerol saline served as positive and negative controls, respectively. A skin test was considered positive when its larger diameter was ≥3 mm than the negative control. The value of the total Immunoglobulin E (IgE) was determined.

The nature and severity of asthma was assessed by the values of forced spirometry: FEV1 (forced expiratory volume in 1 s), FVC (forced vital capacity), PEF (forced expiratory flow), MMEF (maximal mid-expiratory flow) and post-bronchodilator FEV1 improvement; exhaled nitric oxide (FeNO); personal history of occupational asthma or asthma worsened in the workplace; asthma severity and treatment step according to GINA [29]; miniAQLQ quality of life questionnaire (mini asthma quality of life questionnaire) [30], a shorter version of AQLQ (asthma quality of life questionnaire) also validated in Spanish [31]; and the ACT asthma control questionnaire (asthma control test) [32] validated in Spanish [33].

### Genetic analysis

For the molecular analysis, genomic DNA was obtained from whole blood of each individual, by a standard procedure using the automated Maxwell 16 DNA extraction system (Promega, Madison, Wisconsin, USA).

The 1102 bp flanking fragment from the *FLG* gene regions of interest was amplified by PCR using the extracted genomic DNA and FilH1F3 and RPT2P1 primers described by Smith et al. [8]. The determination of these mutations was performed using two methods:

- For the R501X mutation a sequencing study was carried out in a 3100 genetic analyzer (Applied Biosystems, CA, USA) automatic sequencer, using the aforementioned oligonucleotides for PCR reactions. The sequencing study allowed us to analyse this region and detect new SNPs. The Genbank accession number for the reference genomic sequence used for *FLG* was NG_016190.1. The analysis was performed blind with respect to case-status monitoring.
- For the 2282del4 mutation a RFLP analysis by digestion with the *Dra*III restriction enzyme was performed. Results of the respective digestions were visualized in a 1 % agarose gel-red stained gel in TBE buffer. The presence of the mutated allele (deletion of 4 amino acids) involved the appearance of a new restriction target and electrophoresis display of two fragments 962 and 136 pb.

### In silico analysis

The in silico analysis was performed using the following programs:

ChromasLite version 2.01 (Technelysium Pty Ltd, Brisbane, Australia), Vector NTI 10 (Invitrogen, Carlsbad, California, USA), OMIM^®^ (http://www.ncbi.nlm.nih.gov/omim), dbSNP of the NCBI (http://www.ncbi.nlm.nih.gov/snp).

The prediction analysis of polymorphisms functional effect was carried out by online platforms ExPASyProSite (http://www.expasy.org/), SNPeffect 4.0 (http://snpeffect.switchlab.org/) and PolyPhen-2 prediction of functional effects of human nsSNPs (http://genetics.bwh.harvard.edu).

### Statistical analysis

The descriptive, bivariate and multivariate analysis was performed using SPSS version 15.0 (Chicago, Illinois, USA).

Genotype, allele and haplotype frequencies were compared in the study and control populations by the SHEsis online statistical platform (http://analysis.bio-x.cn/myAnalysis.php).

Hardy–Weinberg equilibrium was tested by a χ^2^ test (Pearson’s p value). Bivariate analysis used analysis of variance to compare continuous outcomes across the levels of each genotype. Dichotomous variables were analyzed using χ^2^, Fisher’s exact test, Monte Carlo simulation after 10^4^ iterations and odds ratio test (95 % confidence interval) for comparisons of the distribution of categorical variables. A non parametric test such as Kruskall–Wallis was applied for non normal distributions. Logistic Regression was used to model the effects of multiple covariates on the continuous and dichotomous outcomes. Age and sex were included as potential covariates in multivariate analysis. A p value of less than 0.05 was considered statistically significant. To correct for multiple comparisons Bonferroni correction was applied. Considering that in some cases a much lower p value based on multiple comparison corrective procedures would result in unnecessarily low power, the false positive report probability (FPRP) is provided. The FPRP depends on the prior probability π of a true association of the tested association, the α level or observed p-value, and the statistical power to detect the odds ratio of the alternative hypothesis at the given α level or p value. Statistical power is based on sample size, frequency of the at-risk genetic variant, and the specified odds ratio for the presumed association under the alternative hypothesis [34]. A statistical power near 80 % for α error of 0.05 was considered.

## Results

### Study population

The mean age of the study group was 47.9 ± 17.9 years (36.2 ± 13.2 in AA, 56.9 ± 15.8 in NA and 52.1 ± 17.5 in HC) with significant differences between groups [p < 0.001 between the 3 groups, p = 0.007 between HC and All Asthma group (46.5 ± 17.9 years) and p < 0.001 between AA and NA]. There were 40.5 % men in the study sample (53.3 % in AA, 32.0 % in NA and 34.0 % in HC) with significant differences between groups [p < 0.001 between the 3 groups, p > 0.05 between all asthma group (42.7 %) and HC and p < 0.001 between AA and NA].

In AA, 21.3 % skin tests were positive only to seasonal allergens (pollens), 15.3 % for perennial allergens (mites, moulds and dander) and 65.3 % for both seasonal and perennial allergens. The median number of positive skin tests was 6.0 (interquartile range 4.0). Total IgE mean was 189.6 ± 384.6 IU/mL (379.8 ± 535.8 in AA, 68.6 ± 117.7 in NA and 86.0 ± 235.2 in HC) with significant differences between groups [p < 0.001 between the 3 groups, p < 0.001 between all asthma group (224.2 ± 417.4 IU/mL) and HC and p < 0.001 between AA and NA].

Family and personal history are shown in Table 1. There were significant differences between groups in all of them except in the history of psoriasis and *ichthyosis vulgaris*.

**Table 1:** Clinical characteristics, lung function values and FeNO of the patients included in the study

	All asthmatic patients (n = 300)	AA (n = 150)	NA (n = 150)
Family history of atopy (%)*	120 (40.0 %)	74 (49.3 %)	46 (30.7 %)
Atopic dermatitis (%)*	15 (5.0 %)	14 (9.3 %)	1 (0.7 %)
Contact dermatitis n (%)	73 (24.3 %)	34 (22.7 %)	39 (26.0 %)
Food allergy (%)*	32 (10.7 %)	28 (18.7 %)	4 (2.7 %)
Drug allergy (%)*	58 (19.3 %)	19 (12.7 %)	39 (26.0 %)
Hypersensitivity to NSAID (%)*	34 (11.3 %)	6 (4.0 %)	28 (18.7 %)
Psoriasis (%)	4 (1.3 %)	2 (1.3 %)	2 (1.3 %)
Ichthyosis vulgaris (%)	0 (0.0 %)	0 (0.0 %)	0 (0.0 %)
Lung function values mean ± SD			
FVC (%)*	97.2 ± 16.0	100.9 ± 13.0	93.6 ± 17.9
FEV1 (%)*	93.0 ± 17.5	96.4 ± 14.2	89.7 ± 20.0
FEV1/FVC*	74.7 ± 8.6	76.3 ± 8.0	73.0 ± 8.8
Post-bronchodilator FEV1 improvement (%)	12.81 ± 9.2	12.56 ± 8.5	13.1 ± 9.8
PEF (%)*	95.0 ± 20.7	99.7 ± 18.2	90.3 ± 22.0
MMEF (%)*	63.4 ± 25.7	69.8 ± 21.1	57.1 ± 28.3
FeNO (ppb) mean ± SD*	32.3 ± 30.8	38.0 ± 35.3	26.6 ± 24.2

*AA* Allergic Asthma group, *NA* Non-allergic Asthma group, *NSAID* non-steroidal anti-inflammatory drugs, *FVC* Forced Vital Capacity, *FEV1* Forced Expiratory Volume in 1 s, *PEF* forced expiratory flow, *MMEF* maximal mid-expiratory flow, *FeNO* exhaled nitric oxide

* p < 0.05

Subjects suffering from rhinitis constituted the 87 % of the total asthmatic patients, 94 % in the case of AA and 80 % for the NA group (p < 0.05). In the NA group 52 % of patients had chronic rhinosinusitis. A greater proportion of nasal polyposis (28 vs 6 %) and anosmia (16.7 vs 8 %) was found in the NA group (p < 0.05).

Significant differences were observed in spirometry values (except post-bronchodilator improvement) as well as the value of FeNO. See Table 1. However, no significant differences were found in the value of the miniAQLQ or ACT.

Classifying the severity of asthma according to the GINA guidelines, 2.7 % had intermittent asthma (4.7 % in AA and 0.7 % in NA), 9.0 % mild persistent asthma (12.7 % in AA and 5.3 % in NA), 81.7 % moderate persistent asthma (80.7 % in AA and 82.7 % in NA) and 6.7 % severe persistent asthma (2.0 % in AA and 11.3 % in NA). Significant differences between groups (p < 0.001) were observed.

### R501X and 2282of4 mutations

The genotype and allele frequencies of R501X and 2282del4 mutations can be seen in Table 2. All subjects carried heterozygous mutations. No combined heterozygote patient was identified.

**Table 2:** Genotype frequencies of the 12 SNPs identified in our population

		Total	HC	AA	NA	All asthmatic patients
	n	400	100	150	150	300
R501X	CC	0.98	0.98	0.98	0.99	0.99
	CT	0.02	0.02	0.02	0.01	0.01
	TT	0.00	0.00	0.00	0.00	0.00
2282del4	NN^a^	0.99	0.99	1.00	0.99	1.00
	ND^a^	0.01	0.01	0.00	0.01	0.00
	DD^a^	0.00	0.00	0.00	0.00	0.00
1741A > T	AA	1.00	0.99	1.00	0.97	0.99
	AT	0.00	0.01	0.00	0.03	0.01
	TT	0.00	0.00	0.00	0.00	0.00
1494G > C	GG	1.00	0.99	1.00	1.00	1.00
	GC	0.00	0.01	0.00	0.00	0.00
	CC	0.00	0.00	0.00	0.00	0.00
1560G > A	GG	1.00	0.99	1.00	1.00	1.00
	GA	0.00	0.01	0.00	0.00	0.00
	AA	0.00	0.00	0.00	0.00	0.00
1594C > T	CC	1.00	1.00	1.00	0.99	1.00
	CT	0.00	0.00	0.00	0.01	0.00
	TT	0.00	0.00	0.00	0.00	0.00
1603T > C	TT	1.00	1.00	1.00	0.99	1.00
	TC	0.00	0.00	0.00	0.01	0.00
	CC	0.00	0.00	0.00	0.00	0.00
1607T > A	TT	1.00	1.00	0.99	1.00	1.00
	TA	0.00	0.00	0.01	0.00	0.00
	AA	0.00	0.00	0.00	0.00	0.00
1632C > T	CC	1.00	1.00	1.00	0.99	1.00
	CT	0.00	0.00	0.00	0.01	0.00
	TT	0.00	0.00	0.00	0.00	0.00
1676A > G	AA	1.00	1.00	0.99	1.00	1.00
	AG	0.00	0.00	0.01	0.00	0.00
	GG	0.00	0.00	0.00	0.00	0.00
1747C > A	CC	1.00	1.00	0.99	1.00	1.00
	CA	0.00	0.00	0.01	0.00	0.00
	AA	0.00	0.00	0.00	0.00	0.00
1807G > A	GG	1.00	1.00	0.99	1.00	1.00
	GA	0.00	0.00	0.01	0.00	0.00
	AA	0.00	0.00	0.00	0.00	0.00

*HC* Healthy Controls group, *AA* Allergic Asthma group, *NA* Non-allergic Asthma group

^a^ *N* normal allele, unmutated. *D* deleted allele

In our study we found no association between filaggrin R501X and 2282del4 mutations and the presence of asthma assessed globally or by its two phenotypes. Nor were significant differences found in the personal history for sinonasal disease, spirometry values, FeNO, severity of asthma according to the GINA guidelines, treatment step according to the GINA guidelines, miniAQLQ or ACT.

### New SNPs

An SNP search was performed in the sequenced fragment to study the R501X mutation in the 1.102pb fragment amplified by PCR.

Three novel polymorphisms not previously described were identified for the first time in this genomic region (1594C > T, 1607T > A and 1747C > A). These three polymorphisms located in the exon region also generate changes in the encoded amino acid (see Table 3).

**Table 3:** SNPs found in our population

SNP		Reference^a^	SNP	Protein change	Global minor allele frequency^a^
1494G > C	p.E498D	rs13376095	G/C	Glu → Asp	0.0068
1560G > A	p.P520P	rs369161171	G/A	Pro → Pro	–
1594C > T	p.H532Y		C/T	His → Tyr	
1603T > C	p.S535P	rs74129464	T/C	Ser → Pro	0.0154
1607T > A	p.V536E		T/A	Val → Glu	
1632C > T	p.S544S	rs137997325	C/T	Ser → Ser	0.0020
1676A > G	p.H559R	rs546475787	A/G	His → Arg	0.0004
1741A > T	p.T581S	rs145627745	A/T	Thr → Ser	0.0030
1747C > A	p.H583 N		C/A	His → Asn	
1807G > A	p.V603 M	rs137995883	G/A	Val → Met	0.0010

*SNP* Single-Nucleotide Polymorphism

^a^Obtained from dbSNP (http://www.ncbi.nlm.nih.gov/projects/SNP/)

In addition, in our population another 7 polymorphisms were detected that were previously described but which had not been analysed in asthmatic patients (1494G > C, 1560G > A, 1603T > C, 1632C > T, 1676A > G, 1741A > T y 1807G > A). See Table 3.

SNP 1741A > T was identified in five individuals (1.25 % of our population), the rest only in one (0.25 % of our population). The genotype frequencies of SNP can be seen in Table 2. No subject simultaneously carried more than one variation from the 12 SNPs studied. None of the 15 patients with a history of atopic dermatitis were carriers of any of the 12 SNP studied.

## 1741A > T

SNP 1741A > T (rs145627745) is found in genomic position 1741 and is an adenine-to-thymine transversion. The protein carries a modification in position 581, where the amino acid threonine (Thr) changes to a serine (Ser), so this SNP can also be designated as p.T581S or p.Thr581Ser. This change is present in 5 subjects, 4 of the NA group (2.67 %) and 1 of the HC (1.0 %). The genotype and allele frequencies are reflected in Table 2. No statistically significant differences were found when analysing age or gender differences, indicating the absence of a bias based on such variables.

We identified a statistically significant association between the asthma phenotype and 1741A > T, being the most common mutated allele in patients with non-allergic asthma (genotypic p = 0.044 and allelic p = 0.045). See Table 4. However, no association was identified between 1741A > T and the presence of both overall asthma and asthma worsened in the workplace or occupational asthma.

**Table 4:** Allele and genotype frequencies of 1741A > T SNP in allergic and non-allergic asthma

	Genotype frequency 1741A > T				Allele frequency 1741A > T		
	AA	AT	TT	p	A	T	p
HC (n = 100) vs All Asthma (n = 300)	0.99 (n = 99) 0.99 (n = 296)	0.01 (n = 1) 0.01 (n = 4)	0.00 (n = 0) 0.00 (n = 0)	*0.79*	0.99 (n = 99) 0.99 (n = 296)	0.01 (n = 1) 0.01 (n = 4)	*0.80*
AA (n = 150) vs NA (n = 150)*	1.00 (n = 150) 0.97 (n = 146)	0.00 (n = 0) 0.03 (n = 4)	0.00 (n = 0) 0.00 (n = 0)	*0.044*	1.00 (n = 0) 0.99 (n = 146)	0.00 (n = 0) 0.01 (n = 4)	*0.045*

*HC* Healthy controls group, *AA* Allergic asthma group, *NA* Non-allergic asthma group

* Fisher’s p < 0.05

When analysing the values of forced spirometry we found a statistically significant association between 1741A > T and FVC (p = 0.006), FEV1 (p = 0.020) and PEF (p = 0.002) (see Table 5), confirmed by logistic regression adjusted by sex and age. No statistically significant associations were observed with FeNO.

**Table 5:** Spirometry and exhaled nitric oxide values in patients with non-allergic asthma, according to the 1741A > T SNP

Average per genotype	1741A > T		
	AA (n = 296) mean ± SD	AT (n = 4) mean ± SD	p
FVC (%)*	97.5 ± 15.8	75.3 ± 13.7	0.006
FEV1 (%)*	93.3 ± 17.4	72.9 ± 9.5	0.020
FEV1/FVC	74.8 ± 8.6	69.2 ± 7.3	0.20
Post-bronchodilator FEV1 improvement (%)	12.8 ± 9.2	13.1 ± 5.8	0.94
PEF (%)*	95.5 ± 20.5	63.5 ± 8.0	0.002
MMEF (%)	63.8 ± 25.7	38.9 ± 14.3	0.055
FeNO (ppb)	32.3 ± 30.9	34.0 ± 24.3	0.91

*FVC* Forced Vital Capacity, *FEV1* Forced Expiratory Volume in 1 s, *PEF* forced expiratory flow, *MMEF* maximal mid-expiratory flow, *FeNO* exhaled nitric oxide

* p < 0.05

No statistically significant association in the severity of asthma was observed according to the GINA guideline and 1741A > T, but there were differences in the treatment step according to that guideline (p = 0.004): step one 3.3 vs 0 %, step two 9.0 vs 0 %, step three 56.7 vs 0 %, step four 27.7 vs 1.0 % and step five 2.0 vs 0.3 %, confirmed by logistic regression adjusted by sex and age.

The analysis using the ExPASyProsite platform of the functional change produced by this SNP indicates that it is located exactly on a myristoylation site that could have an impact on the protein’s activity. According to the analysis conducted by SNPeffect this SNP does not affect the amyloid, binding to chaperones or aggregation tendency. In the study of functional prediction by PolyPhen-2 prediction of functional effects of human nonsynonymous SNPs we observed a score of 0.083 (sensitivity: 0.93; specificity: 0.85).

Considering the scarce representation of *FLG* mutant alleles a combined variable using all the nonsynonymous SNPs was created. Significant association was detected between mutant alleles and FVC (p = 0.011), FEV1 (p = 0.001), FEV1/FVC (p = 0.019), PEF (p = 0.002), MMEF (p = 0.007) as well as lower total IgE levels (p = 0.028), see Table 6. These findings are also found in the non-allergic group for FVC (p = 0.002), FEV1 (p = 0.002), PEF (p = 0.014) and MMEF (p = 0.029) and in the allergic group only for FEV1/FVC (p = 0.034). This was confirmed by logistic regression adjusted by sex and age except total IgE.

**Table 6:** Associations of combined variable using all the nonsynonymous SNPs

	Mutant alleles	No mutant alleles	p
FVC (%) (n = 15 vs 285)	87.9 ± 19.2	97.7 ± 15.7	0.011
FEV1 (%) (n = 15 vs 285)	80.5 ± 19.1	93.7 ± 17.1	0.001
FEV1/FVC (n = 15 vs 285)	70.6 ± 9.7	74.9 ± 8.5	0.019
PEF (%) (n = 15 vs 285)	81.3 ± 22.5	95.8 ± 20.4	0.002
MMEF (%) (n = 15 vs 285)	48.8 ± 21.7	64.3 ± 25.7	0.007
Total IgE (IU/mL) (n = 20 vs 380)	120.3 ± 255.7	193.7 ± 390.6	0.028

*FVC* Forced Vital Capacity, *EV1* Forced Expiratory Volume in 1 s, *PEF* forced expiratory flow, *MMEF* maximal mid-expiratory flow, *FeNO* exhaled nitric oxide, *IgE* Immunoglobulin E

## Discussion

In this study, the R501X and 2282del4 mutations were not identified as being associated with any of the patients’ clinical and biological characteristics. It is noteworthy that the allele frequencies of these mutations in our population were lower than those obtained in European population studies [10, 21]. This could be due to the inclusion criteria of the study, but may also indicate particular aspects of the Spanish population.

In this study we identified for the first time three polymorphisms that had not been previously described in the filaggrin gene (1594C > T, 1607T > A and 1747C > A). It is very interesting to note that one of the polymorphisms identified in this work, 1741A > T, showed an association with non-allergic asthma and with a greater severity of it. In our series, none of the patients with personal history of atopic dermatitis were carriers of any of the 12 identified SNPs.

Mutations in the filaggrin gene have shown great importance in *ichthyosis vulgaris* [8] and atopic dermatitis [9–19], but no clear relationship to asthma has been deduced [10–26]. There is a considerable controversy about the possible association with asthma and it is unclear whether the association detected in some studies is due to the presence of asthma or the influence of eczema or atopic dermatitis in individuals who have asthma associated with these skin diseases, as two meta-analyses concluded [27, 28].

We found statistically significant differences in SNP 1741A > T when analysing the spirometry values: patients carrying the T allele presented lower FVC, FEV1 and PEF values than those carrying the A allele. We also found statistically significant differences when analysing the treatment step required by patients according to GINA guidelines, requiring a step higher than 3 the patients carrying the T allele. Some mutations in the *FLG* gene have been associated with the presence of asthma (in particular allergic asthma and asthma with atopic dermatitis). Despite having been described in the databases this SNP has not been studied in populations of patients with atopic dermatitis or asthma. Interestingly, in our population it was associated to non-allergic asthma. Gene expression studies indexed in GeneCards^®^ (http://www.genecards.org) show that the *FLG* gene expression was observed in lung and bronchial mucosa, despite of the lack of protein detection in such mucosa [7]. In addition, filaggrin is expressed in oral mucosa, where it is assumed that it also contributes to the barrier function [3, 4]. It has also been shown to be expressed in the nasal vestibule up to the transitional epithelium [5] and, very recently, in the nasal mucosa [6]. The link between upper and lower airways and the relation between asthma and nasal polyposis are well known [35] and this could be related to the association found between 1741A > T SNP and non-allergic asthma, as 2 of the 4 non-allergic asthmatic patients carrying the SNP had chronic rhinosinusitis. Nevertheless, in our study none of them had a personal history of nasal polyps, although this entity is frequently infradiagnosed. The epithelium plays an important role in the inception and development of asthma, particularly in non-allergic asthma, through the activation of type 2 innate lymphoid cells (ILC2) [36] and SNPs in filaggrin could compromise skin barrier function, increase penetration of allergens, reduce inflammatory threshold levels to skin irritants and allergens and increase the risk of sensitization [37]. It is tempting to speculate that microbes, pollutants, tobacco smoke or other factors could easier cross the epithelial barrier and induce the inflammatory process in allergic asthma but maybe also in non-allergic asthma.

This SNP generates a change from threonine to serine in the filaggrin protein. While the predicted level of pathogenicity for this SNP is not very high, we have identified that this change is found in a pattern of myristoylation of the protein. Myristoylation of proteins occurs when there is a transfer of a myristoyl group to the glycine amino group of a specific protein sequence, using Myr-CoA as substrate. It is assumed that myristoylation is an irreversible process that occurs co-translationally. In fact, myristoylation of proteins increases their hydrophobicity and thus could have a functional impact not described to date. More analysis of the protein produced in patients carrying the T allele are needed.

Nine other polymorphisms have been described; three of them (1594C > T, 1607T > A and 1747C > A) have been described for the first time in this study. Nevertheless, no clinical associations were identified, perhaps due to the low frequency of occurrence, at least in our population. Considering the scarce representation of FLG mutant alleles a combined variable using all the nonsynonymous SNPs was created. This approach has been used in the field of *FLG* mutations due to their low frequency. A significant association was detected between carrying mutant alleles and worse respiratory function values in non-allergic asthma.

We think it is important to point out that our study was prepared from the outset to assess the influence of these mutations and SNPs in the filaggrin gene in asthma and potential importance of atopy in this association. Therefore, we included asthmatic patients divided into two groups with well-differentiated AA and NA phenotype and solid clinical classifications, and a control group rigorously selected without personal or family history of atopy. Our results are consistent with several studies in other populations that question the association of mutations in the *FLG* gene and asthma [10, 14, 17, 20], but interestingly in our study the association seems to be with non-allergic asthma without eczema or atopic dermatitis. In this sense, it could be interesting to interrogate possible differences regarding filaggrin expression in the bronchial mucosa between allergic and non allergic asthma to assess implication of SNPs in the filaggrin gene.

In our population, we detected no association for any of the variants of the *FLG* gene analysed with atopic dermatitis or with any other clinical variable, possibly because our patients, unlike those of other studies, were selected due to presenting asthma and not dermatitis as initial clinical variable, making them a distinct population. In this respect this result would indicate the existence of distinct genetic associations for both diseases and again could explain the contradictory results obtained in the literature.

In conclusion, in our population we found no significant differences with null-mutations R501X and 2282del4, but we did find significant differences with SNP previously described in the *FLG* gene but which has not been studied in patient populations. In addition, we have first identified three polymorphisms that had not been previously described in *FLG* gene (1594C > T, 1607T > A and 1747C > A).

## Conclusions

In the association study of genetic variants of the FLG gene in our population the 1741A > T polymorphism seems to be associated with non-allergic asthma.

## Abbreviations

95 % CI: 95 % confidence interval
AA: allergic asthma
ACT: asthma control test
AQLQ: asthma quality of life questionnaire
ARIA: allergic rhinitis and its impact on asthma
EDC: epidermal differentiation complex
EPOS: European position paper on rhinosinusitis and nasal polyps
FeNO: exhaled nitric oxide
FEV1: forced expiratory volume in 1 s
FPRP: false positive report probability
FVC: forced vital capacity
GINA: global initiative for asthma
HC: healthy controls
IgE: immunoglobulin E
miniAQLQ: mini asthma quality of life questionnaire
MMEF: maximal mid-expiratory flow
NA: non-allergic asthma
NSAIDs: non-steroidal anti-inflammatory drugs
PEF: forced expiratory flow
SNP: single-nucleotide polymorphism
